# High level of self-control ability in a small passerine bird

**DOI:** 10.1007/s00265-018-2529-z

**Published:** 2018-06-26

**Authors:** Emil Isaksson, A. Utku Urhan, Anders Brodin

**Affiliations:** Department of Biology, Ecology Building, Lund University, S-223 62 Lund, Sweden

**Keywords:** Self-control, Cognition, Brain volume, Transparent cylinder test, Great tit

## Abstract

**Abstract:**

Cognitively advanced animals are usually assumed to possess better self-control, or ability to decline immediate rewards in favour of delayed ones, than less cognitively advanced animals. It has been claimed that the best predictor of high such ability is absolute brain volume meaning that large-brained animals should perform better than small-brained ones. We tested self-control ability in the great tit, a small passerine. In the common test of this ability, the animal is presented with a transparent cylinder that contains a piece of food. If the animal tries to take the reward through the transparent wall of the cylinder, this is considered an impulsive act and it fails the test. If it moves to an opening and takes the reward this way, it passes the test. The average performance of our great tits was 80%, higher than most animals that have been tested and almost in level with the performance in corvids and apes. This is remarkable considering that the brain volume of a great tit is 3% of that of a raven and 0.1% of that of a chimpanzee.

**Significance statement:**

The transparent cylinder test is the most common way to test the ability of self-control in animals. If an animal understands that it only can take food in the cylinder from the cylinder’s opening and controls its impulsivity, it passes the test. A high level of self-control has been demonstrated only in cognitively advanced animals such as apes and corvids. Here, we demonstrate that the great tit, a small song bird that is very good at learning, performs almost in level with chimpanzees and ravens in this test.

**Electronic supplementary material:**

The online version of this article (10.1007/s00265-018-2529-z) contains supplementary material, which is available to authorized users.

## Introduction

Self-control, or the ability to inhibit impulses that ultimately are counter-productive, is a fundamental cognitive skill that is necessary for processes such as decision-making and planning. It is important both for animals and humans since rational behaviour would not be possible without it. Without self-control, animals and humans would be at the mercy of impulses, not being able to choose how to behave (e.g. Freud 1930; Diamond 2013). Inhibitory control is an executive cognitive function that is thought to play an important role in complex cognitive abilities (MacLean et al. 2014; Kabadayi et al. 2016; Vernouillet et al. 2016).

A common test of self-control in animals is the transparent cylinder test. In this test, the animal is first trained to take food from inside an opaque cylinder that is open in both ends. After training, the opaque cylinder is replaced by a transparent one that is positioned perpendicularly to the direction of the animal’s approach. The animal passes the test if it moves to either opening of the cylinder and takes the food from inside it. If it tries to take the food through the transparent wall, it fails the test. This test requires inhibitory skills to overcome immediate motor responses and is thus a test of motor self-regulation. As the term motor self-regulation requires only that an animal can inhibit a movement, we will use the more descriptive blanket term “self-control” henceforward when we discuss the cylinder task, even though some authors, e.g. Beran (2015) and Kabadayi et al. (2016), argue against this. Another way of testing self-control in animals is to use delay of gratification. In this, an animal has to decline a small immediate reward for a future larger one. Goffin cockatoos *Cacatua goffini*, ravens *Corvus corax*, and carrion crows *Corvus corone corone* could refrain from an immediate small reward for a later larger one, but only for a few minutes (Dufour et al. 2012; Auersperg et al. 2013; Kabadayi and Osvath 2017).

MacLean et al. (2014) compared self-control in the cylinder task in 36 species of mammals and birds and found that the strongest predictor of performance is absolute brain volume. Recently, Kabadayi et al. (2016) showed that three species of corvids; ravens, New Caledonian crows *C*. *moneduloides*, and jackdaws *C*. *monedula*, performed on the same level as the great apes, 90–100%, even though they have much smaller brains. Adding data on these corvids to the data set of MacLean et al. made Kabadayi et al. conclude that not only absolute, but also relative brain volume was a reliable predictor of performance in the cylinder task in birds. The average performance over the first ten test sessions has been the measure in these comparisons between animal species. A level of 100% means that all individuals performed correctly already at the first attempt and a level of 90% that they on average performed correctly in nine out of the ten first attempts. It should be noted that in the cylinder task, the reward is almost instant. Self-control may be more difficult if there is a long time delay before gratification (e.g. Dufour et al. 2012; Auersperg et al. 2013; Kabadayi and Osvath 2017).

Before being exposed to the transparent cylinder test, the subjects are usually given ample opportunities to interact with transparent objects. The corvids in Kabadayi et al. (2016) were raised with transparent objects being present in their home cages, for example windows and open-ended pieces of plastic bottles. The rationale for this is to maximise the probability that the cylinder test becomes a test of impulse control rather than a test of the ability to learn to understand transparency.

The great tit *Parus major* is a small passerine bird with a body mass around 17 to 18 g. It is known to be innovative and especially good at learning new foraging tasks (Sasvári 1979; Brodin and Urhan 2014; Aplin et al. 2015). Considering this, we see it as likely that this species should be able to pass the cylinder test even though its brain size is only around 0.42 cm^3^ (Healy and Krebs 1996). This is less than 3% of that of a raven, around 8% of that of a jackdaw, and slightly below 6% of that of a New Caledonian crow (Kabadayi et al. 2016).

Our aim with this study was to test the great tit in the transparent cylinder test and investigate how our great tit data relates to the suggestion by MacLean et al. (2014) that absolute brain volume predicts the ability of self-control.

## Material and methods

### Subjects

From late August 2016 to March 2017, we captured 36 great tits in and around Höör (55.93 N, 13.32 E) and Lund (55.70 N, 13.19 E) in southernmost Sweden, using mist nets and playback recordings of great tit song. After removing the birds from the nets, we kept them in individual cotton bags while we transported them to an indoor animal facility at the Department of Biology, Lund University. The transport from the capture sites to this facility took a maximum of 30 min. In the laboratory, we transferred the birds to individual cages measuring 60 cm × 60 cm × 40 cm. We kept the birds in these cages during the rest of their stay in the laboratory except for during the experimental sessions. Since great tits are a social species, we had placed the individual cages pairwise on shelves so that each bird could have visual and vocal contact with at least one nearby other individuals.

In their home cages, the birds had ad libitum access to water and food. We enriched the water with a vitamin mixture for birds (Allvitamin för burfåglar, IMAZO). As the main food source, we provided a mixture of sunflower seeds, peanuts, and hemp seeds. We also provided suet cakes that consisted of animal fat and nuts. If a bird initially appeared not to eat sufficiently, we additionally provided living food in the form of mealworms *Tenebrio molitor* and zofobas larvae *Zophobas morio*, since live food increases great tits’ motivation to eat. This live food was given at irregular intervals to the birds. Before we started to train the birds, we allowed them to get accustomed to the lab for a minimum of 2 days. During the whole experiment, all birds appeared to be in good nutritional condition.

When we had completed all sessions on a bird, we released it at the same location where we originally had captured it. Before we released a bird, we inspected it and made sure that it was in good condition. We performed the experiments and kept the birds under permit M-213-11 from the Malmö-Lund regional ethical permit board.

### The facility

We kept the birds and performed the experiments in two especially designed bird rooms that measure 5 m × 2.6 m × 3 m. These are equipped with computer controlled temperature and light regimes. We kept the temperature constant at 14 °C and adjusted day length to approximately match outdoor conditions (between 10/14 h—light/dark and 8/16 h—light/dark). The lights have a daylight spectrum and a 1-h dimming function that simulates dawn and dusk.

Before each experimental or training session, we allowed a bird to move from its home cage to a special experimental cage. During both training and experimental sessions, the birds and the experimenter were in separate compartments of the room. In order to minimise disturbance of the birds, there was a screen of dark smoke-coloured glass between the experimenter’s compartment and the birds. As the experimenter’s side of the screen was dark and the birds’ side lit up, it was possible to observe the birds without being seen by them.

Great tits are very good observational learners (Sasvári 1979; Cole and Quinn 2013; Brodin and Urhan 2014; Aplin et al. 2015), so to prevent the birds seeing other individuals doing the test, we kept the home cages of the birds in a part of the lab from which they could not see the experiments.

### The experimental cage

The experimental cage was a standard home cage (60 × 60 × 40 cm) that we had equipped with a special experimental box, measuring 36 × 21 × 25 cm. The box was made of MDF boards with an open side towards the cage and a transparent Plexiglas window on the side facing the experimenter. Between the cage and the box, there was a slide door that could be opened or closed by pulling a string from the experimenter’s position. This made it possible to either keep the birds in the cage or give them access to the box. At the bottom of the box, we had mounted a small rotatable platform that the experimenter could turn in either direction by pulling one of two strings. The birds had full view of the inside of the box from the cage. We recorded all training and experimental sessions with a Sony Action Cam HDR-AS200VT. We designed both training and experimental methods so that they should be as similar as possible to MacLean et al. (2014) and Kabadayi et al. (2016) to facilitate comparisons with these studies.

### Experimental procedure

We used three birds to test and develop the methods and the remaining 33 in the experiment. It is assumed that animals need to habituate to transparency before they can learn to pass the cylinder test (Kabadayi et al. 2016). In order to do this, we assigned the birds to one of three experimental groups, 11 in each. For group 1, we mounted a transparent small wall measuring 17 × 17 cm in the home cage. We positioned it perpendicularly to the wall, at the bottom of the cage. This group can be seen as having experience with transparency in a general sense. For group 2, we introduced a similar (but different) transparent cylinder as the one used in the experiment at the bottom of the home cage (with no food in it). This group can be seen as having specific experience of a transparent cylinder-shaped object. The habituation cylinder had the same outer diameter as the test one but was longer and had a smaller inner diameter. Because of this, it was hard for the birds to stick their heads into it in the same way as in the test cylinders. The remaining 11 birds in group 3 served as control group with no previous experience of transparency. We inserted the transparent habituation objects for groups 1 and 2 in their home cages 2 days before we started the training sessions. Groups 1 and 2 can be called transparency-experienced and group 3, transparency-naïve. Group two can be called cylinder-experienced, whereas groups 1 and 3 together would be cylinder-naïve.

Before each training and experimental session, we positioned the bird’s home cage next to the experimental cage. A wide but short plastic tube connected the doors between the two cages so that the bird could move between the cages when the doors were open. When the bird moved over to the experimental cage (which they always did spontaneously), we closed the door to the experimental cage. This procedure made it possible for us to minimise handling of the birds, something that is stressful to them. For great tits, live food, such as mealworms, is very attractive. In order to ensure that the birds were motivated, we made sure that they did not get any live food before training and experimental sessions. After the first training session (with the opaque cylinder), we used rewards consisting of cut pieces of mealworms, approximately 5 mm long, in order to minimise the risk that the birds would be satiated during consecutive sessions.

#### Training sessions

During training sessions, we presented the reward in the centre of an opaque plastic cylinder that was open at both ends. We had positioned the opaque cylinder on the rotatable platform in the centre of the experimental box. The cylinder was 8.5 cm mm long with an outer diameter of 3.5 cm meaning that a great tit easily could reach the reward from both ends of the cylinder. The birds could see the cylinder from the cage but not its content as the cylinder’s openings were perpendicular to the direction of the bird. We started the first training session by rotating the platform 90° so that the bird could see the mealworm inside the cylinder from its position in the cage. After 30 s, we rotated the cylinder back to its original position. We then opened the slide door between the cage and the box so that the bird could enter the box. If a bird managed to retrieve the reward within 6 min, we considered this as a successful trial, although we allowed the bird to stay in the cage until it had taken the reward. We rotated the cylinder only in the first training session and not in consecutive training or test sessions. After a bird had succeeded to retrieve the reward within 6 min in five consecutive sessions, we allowed it to proceed to the test trials.

#### Test sessions

After the training sessions, we replaced the opaque cylinder with a transparent one of the same size. We started the test sessions 1 day after a bird successfully had completed training. We conducted a total of ten test trials on two consecutive days, meaning that each individual was tested in five sessions in 1 day. Between each such session, we allowed the birds a 20-min rest. This reduced the risk that the bird would become satiated and lose motivation to take additional rewards. We placed half a mealworm in the centre of the cylinder and positioned it in the same perpendicular angle relative to the direction of the bird as the opaque cylinder in the training sessions. A bird in the experimental cage would then have full view of the tube and the reward inside it. After a delay of 10 to 30 s depending on how fast the bird spotted the mealworm, we opened the door to the box and allowed the bird to enter it. If the bird’s first manoeuvre was to peck at the transparent wall of the cylinder, we considered this as a failed attempt. If it instead moved to the opening of the cylinder and took the reward from inside it without touching the wall, we counted this as a successful attempt.

We concluded all ten sessions for all birds regardless of whether they had passed the test before session 10. This means that a bird could fail after previously having passed in several subsequent sessions. To test if birds that failed in session 10 still knew how to pass the test, we kept those birds in their home cages for 10 days and then made a follow-up test, consisting of one single session. In accordance with previous studies (MacLean et al. 2014; Kabadayi et al. 2016), we use the average performance over the ten first sessions as the representative measure of performance in the test.

It was not possible to record data blind as we tested one focal, colour-banded individual at a time in a special experimental device.

### Statistical analyses

To compare time spent before taking the reward over sessions we used the repeated measures ANOVA in Statistica’s GLM module. We made the tests of percentages on arcsine square root transformed proportions and the tests of time measures of logged values. We made the latter transformation to homogenise variances and normalise data. To compare performance between categories of birds (males/females, cylinder experienced/cylinder-naïve), we used two-sample *t* tests. For paired comparisons, we used paired *t* tests. All tests are two-sided. As dispersion measure, we used 95% confidence intervals.

#### Data availability

All data generated or analysed during this study are included in this published article and its supplementary Excel file.

## Results

**Fig. 1:**
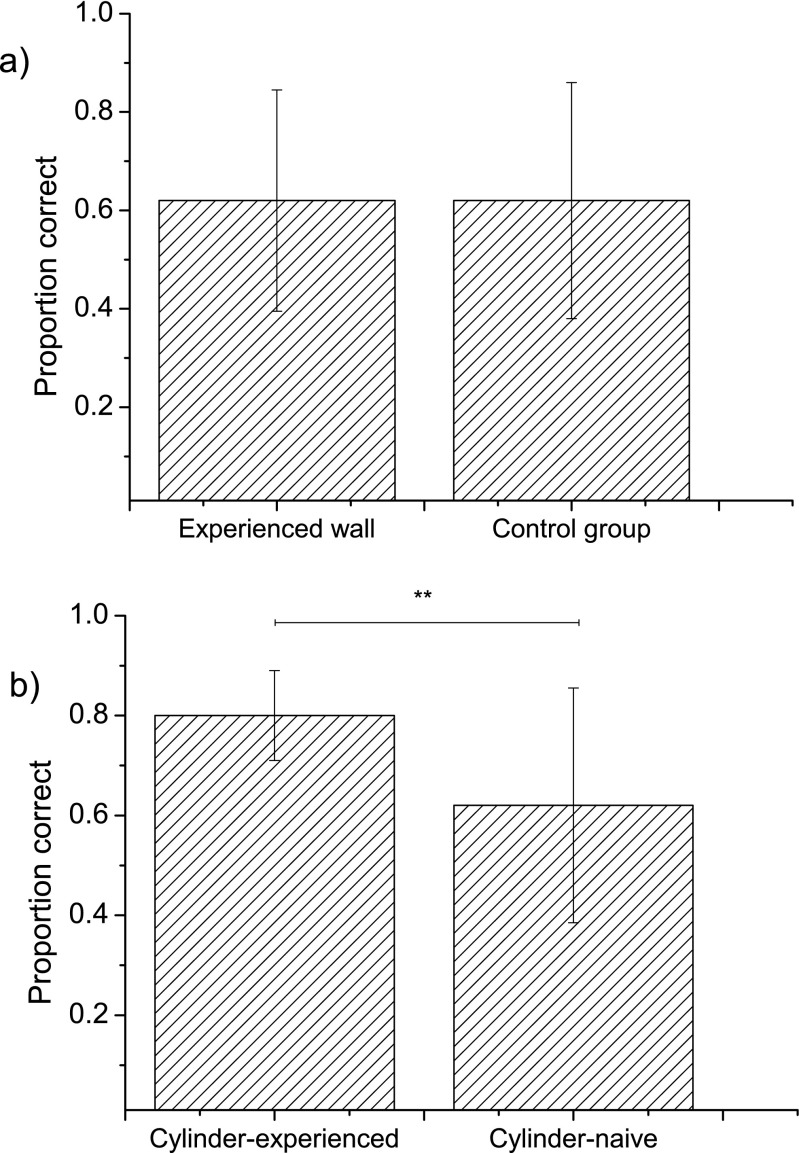
The average proportion of correct attempts in ten sessions in **a** group 1 (birds that had experience of a transparent wall) versus the control group and **b** group 2 (the birds with experience of a transparent cylinder) compared to the pooled groups from **a** (wall + control = “cylinder-naïve”). The error bars are 95% confidence intervals

As the performance of the birds in group 1 (experience of transparent wall) was indistinguishable from that of those in the control group (Fig. 1a), we pooled the data for these 22 birds and call them “cylinder-naïve”. The mean performance of the cylinder-naïve birds in ten tests was 61.4 ± 6.6%. For the females in this group, the mean performance was 57.3 ± 11.8% and for the males 65.5 ± 6.0%, a non-significant difference (*t* = 1.18, *n* = 22, *P* = 0.25). The performance of the cylinder-experienced birds (80 ± 6.8%) was significantly higher than that of the cylinder-naïve birds (*t* = 2.95, *n* = 11 and *n* = 22, *P* = 0.0064, Fig. 1b). The performance of the cylinder-experienced females was 85 ± 6.7% (*n* = 6) and of the cylinder-experienced males was 74 ± 10.0% (*n* = 5), also a non-significant difference (*t* = 1.85, *P* = 0.10). The cylinder-experienced birds also solved the task much quicker 5.1 ± 3.8 s than the cylinder-naïve ones 38.7 ± 32.6 s, a significant difference (*t* = 5.8, *n* = 11 and *n* = 22, *P* < 0.001).

**Fig. 2:**
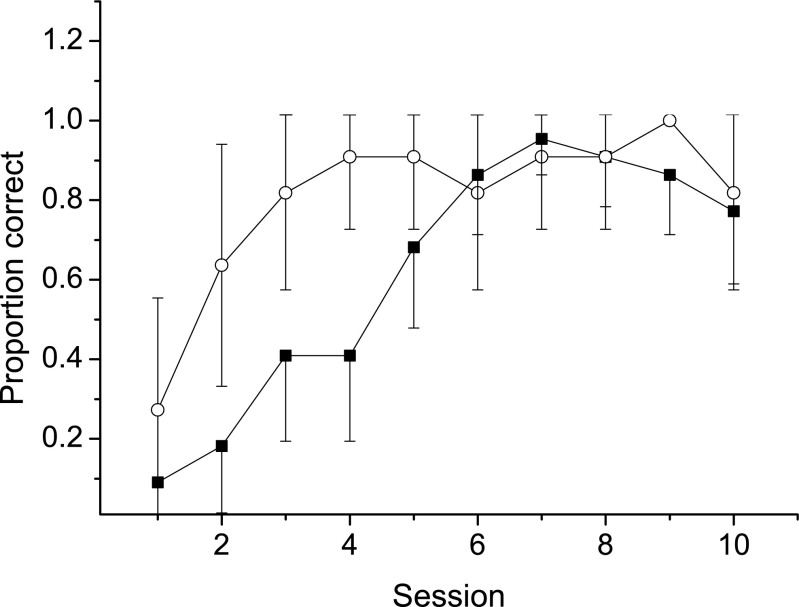
The average proportion correct attempts for the birds in Fig. 1b over ten sessions. The filled squares show the cylinder-naïve birds. The open circles show the cylinder-experienced birds. The error bars 95% confidence intervals

The performance of the cylinder-naïve birds increased at a rather even rate until session 7 when all individuals had passed the test (Fig. 2, filled squares). One bird passed the test in session 6 but failed in session 7, making the average performance 95% rather than 100% in session 7. Three of the 11 cylinder-experienced birds passed the test already in their first attempt (27%, represented by the first open circle in Fig. 2). The others required only one to two sessions to pass the test (Fig. 2, open circles). In both groups, performance tended to decrease somewhat during the last sessions as some birds failed that previously had passed the test. All these birds, however, passed the single follow-up test we made 10 days later. The time that the birds spent to get the reward decreased over sessions (*F*_9,32_ = 18.9, df = 32, *P* < 0.001).

## Discussion

The birds that had experience of transparence in the form of a small glass wall in their home cage did not benefit from this as their performance was almost identical to that of the control group. The birds that had an experience with a transparent cylinder, on the other hand, learned the task quicker which gave them a higher mean performance. An average success of 61 (cylinder-naïve birds) or 80% (cylinder-experienced birds) in the first ten sessions positions the great tit higher than most other birds but below the highest ranking corvids (MacLean et al. 2014; Kabadayi et al. 2016). According to MacLean et al. (2014), animals with small brains should not perform well in this test. MacLean et al. (2014) do not say whether their animals had previous experience with transparency or not. Kabadayi et al. (2016) say that all their birds had extensive experience of transparency from enrichment and experiments. Our results show that the amount of previous experience of a transparent cylinder may have a large impact on the result as a mean performance of 80% is very different from 61%. As the animals in previous transparent cylinder task experiments have had constant exposure to transparent cylinder like objects (Kabadayi et al. 2016), the 80% performance by the cylinder-experienced birds should be the most representative figure in comparison with other studies. If the cylinder test can be considered to be evidence of self-control, we have thus demonstrated such ability in the great tit, with a mean performance of 80% over ten sessions.

It is interesting that experience with a transparent wall did not improve performance in the test. It seems as great tits need experience with round, transparent object in order to perform optimally in the cylinder task. Even if they did not stick their heads into the habituation cylinder, they could peck at its wall and learn that the cylinder’s interior was not accessible through its wall. Birds are able to categorise shapes (Delius 1992), and it is possible that it is easier to generalise from one type of transparent cylinder to another than from a square-shaped window to a cylinder.

A performance of 80% is very high for a bird of this size with a brain that measures 0.44 cm^3^ (Healy and Krebs 1996). Other similar-sized birds that have been tested in the cylinder task are the song sparrow *Melospiza melodia* (performance 26.5%, brain volume 1.06 cm^3^), swamp sparrow *Melospiza georgiana* (performance 26.1%, brain volume 0.81 cm^3^), and zebra finch *Taeniopygia guttata* (performance 52.2%, brain volume 0.44 cm^3^) (MacLean et al. 2014). Only three species of corvids (Kabadayi et al. 2016) and the apes (MacLean et al. 2014) perform better than the great tit of the 36 species of mammals and birds tested to date. Of these, the chimpanzee (brain volume 368 cm^3^) and the raven (brain volume 14.52cm^3^) perform at 100%. With the inclusion of three corvid species, Kabadayi et al. (2016) showed that relative (or residual) brain volume also was a reliable predictor of performance in the cylinder task in birds. We feel that we have strengthened the argument of Kabadayi et al. (2016) with the inclusion of our data on the great tit as it has a very small brain compared to other high-performing species. The brain volume of a great tit is only 3% of that of a “large-brained” bird as a raven and around 0.1% of that of a chimpanzee.

It could be noted that the brain volume measurements in MacLean et al. (2014) and Kabadayi et al. (2016) were done as endocranial volume estimates, a rather crude method. Brain volume in the great tit was measured as telencephalon volume by Healy and Krebs (1996) with microscopy. This should not be a problem, however, as endocranial volume estimation appears to be a reliable method (Iwanyuk and Nelson 2002). If estimations by these methods should differ, the difference should be small and not affect comparisons notably. There could also be other problems when comparisons like this are made between many species. Chimpanzees and ravens perform on a 100% level, meaning that they actually hit the ceiling of the test (i.e. that the test is too simple for them). Finally, as mentioned above, some species may have extensive experience of transparent, cylinder-like objects whereas other may have none at all. According to our results, it may not be meaningful to compare animals that differ much in this respect. If our great tits would have had transparent cylinder-like objects as permanent enrichment in their home cages, like the corvids in Kabadayi et al. (2016), it is possible that they would have performed at an even higher level than 80%. This was not possible for us to test since we used wild-caught birds.

We do not question the claim by MacLean et al. (2014) that absolute brain volume might work as a predictor for the mammals that were included in their data set, but believe that it is not a useful measure in comparisons between birds and mammals. These two classes differ in several respects when it comes to brain morphology. Perhaps, the most important one is that the density of neurons in the avian brain is much higher than in the mammalian brain (Olkowicz et al. 2016). Herculano-Houzel (2017) suggests that the number of neurons in the parts of the brains involved in cognitive processing could be the most relevant measure in comparisons of species from different taxa. Anyway, a higher density of neurons in the avian brain seems logical if one considers the weight constraints imposed on birds due to flying (Emery 2006).

Great tits may benefit from a high level of impulse control also in natural foraging situations. Like other parids, they are very cautious when approaching a food source such as a feeder. An airborne predator such as a sparrowhawk *Accipiter* sp. may appear suddenly from any direction. A great tit approaching a feeder will frequently turn around when it is close to the feeder and return into the cover that it came from. Compared to other parids, great tits are more prone to explore novel objects, such as human-made devices (Adamová-Ježová et al. 2016). It must be beneficial to inspect novel objects visually before perching and pecking at them.

Somewhat unexpectedly, the mean performance decreased during the last sessions. One possible reason for such “late failures” could be that the birds lost motivation, for example if the perceived value of the reward decreased. A second possibility is that the birds pecked at the cylinder wall even though they “knew” that they needed to move to the opening of the tube to get the reward. Regardless of which of these explanations that is correct, all birds, including the ones without experience of a transparent cylinder, were able to solve the problem by session 7.

## General conclusions

In conclusion, all individuals showed evidence of inhibition, which is fundamental for self-control, in seven sessions or less. Experience with a transparent cylinder-like object was important whereas a more general experience of transparency (the wall) had no effect. A few birds made an error after learning the task, but the individuals that failed in the last session succeeded in a follow-up test after a 10-day’s retention interval. The most remarkable result in our study was that an animal with such a small brain as a great tit can perform almost on level with corvids and apes in a cognitive test of this type. Finally, relative brain size is probably a better predictor of self-control than absolute brain size in birds.

## Electronic supplementary material

ESM 1 (XLSX 43 kb)
